# Rumen-Protected Choline Improves Metabolism and Lactation Performance in Dairy Cows

**DOI:** 10.3390/ani14071016

**Published:** 2024-03-27

**Authors:** Fábio Soares de Lima, Manoel Francisco Sá Filho, Leandro Ferreira Greco, José Eduardo Portela Santos

**Affiliations:** 1 Department of Population Health and Reproduction, University of California Davis, Davis, FL 95616, USA; falima@ucdavis.edu (F.S.d.L.); manoel.filho@altagenetics.com (M.F.S.F.); 2 Department of Animal Sciences, University of Florida, Gainesville, FL 32611, USA; leandro.greco@kemin.com

**Keywords:** dairy cow, fatty liver, milk yield, rumen-protected choline

## Abstract

**Simple Summary:**

Choline is a nutrient that plays a vital role in cell structure and functions as a component of phospholipids. In dairy cows, particularly during early lactation, there is often a shortage of essential amino acids such as methionine, which can impact the production of cell components such as phosphatidylcholine. A shortage of choline can further reduce phospholipid availability that might lead to an imbalance in lipid metabolism, resulting in excessive fat accumulation in the liver and potential health issues. To address this, we have explored whether supplementing choline in a rumen-protected form (RPC) helps reduce fatty liver. While some studies suggest that choline supplementation reduces triacylglycerol in the liver prepartum, the results have been inconsistent with postpartum cows. In the present experiment, we investigated the effects of supplementing dairy cows with RPC before and/or after calving on metabolism and lactation performance. Our findings revealed that feeding cows with RPC improved milk production, reduced hyperketonemia (a condition associated with elevated concentrations of ketone bodies in blood), and minimized hepatic lipidosis (excessive fat accumulation in the liver).

**Abstract:**

Choline is required for the synthesis of phosphatidylcholine, an important constituent of lipoproteins. Early lactation cows presumably synthesize insufficient phosphatidylcholine, and choline supplementation in a rumen-protected form might benefit metabolism and lactation. The objectives of this study were to determine the effects of feeding rumen-protected choline (RPC) on lactation and metabolism in dairy cows. In experiment 1, 369 nulliparous and parous Holstein cows housed in four pens per treatment were fed 12.9 g/day of choline as RPC from 25 days prepartum until 80 days postpartum. In experiment 2, 578 nulliparous cows housed in five pens/treatment were fed 12.9 g/day of choline as RPC in the last 22 days of gestation only. In both experiments, blood was sampled and analyzed for concentrations of nonesterified fatty acids (NEFAs) and glucose at 1, 14, and 21 days postpartum and of choline at 1 and 14 days postpartum. Blood from all cows was sampled and analyzed for concentrations of β-OH butyrate (BHB) at 1 and 14 days postpartum. Cows with BHB > 1.2 mmol/L were classified as having hyperketonemia. Hepatic tissue was collected from 46 cows from the eight pens in experiment 1 at 9 days postpartum and analyzed for concentrations of glycogen and triacylglycerol. Milk yield and components were measured for 80 days postpartum in experiment 1, whereas only milk yield was measured in experiment 2. The pen was the experimental unit of analysis. Supplementing RPC tended to increase dry matter intake (DMI) prepartum in experiments 1 and 2 and postpartum in experiment 1. Feeding cows with RPC increased yields of 3.5% fat-corrected milk (42.8 vs. 44.8 kg/day), energy-corrected milk (38.5 vs. 40.3 kg/day), milk fat (1.52 vs. 1.61 kg/day), and true protein (1.16 vs. 1.21 kg/day) in experiment 1. Milk yield tended to be greater with RPC (26.4 vs. 27.4 kg/day) in experiment 2. Supplementing RPC increased plasma choline concentrations on day 14 postpartum in experiment 1 (3.32 ± 0.27 vs. 4.34 ± 0.28 µM) and on day 1 in experiment 2 (3.35 ± 0.16 and 13.73 ± 0.15 µM). Treatment did not affect the concentrations of glucose, NEFAs, or BHB in plasma, but the incidence of hyperketonemia was less in multiparous cows fed RPC than those fed the control in experiment 1. Feeding cows with RPC reduced hepatic triacylglycerol content and tended to reduce the ratio of triacylglycerol to glycogen and the risk of hepatic lipidosis in cows in experiment 1. The concentrations of hepatic triacylglycerol on day 9 postpartum were inversely related to those of choline in plasma on day 1 postpartum. Feeding cows with RPC improved lactation and metabolism, but more benefits were noted when it was fed before and after calving.

## 1. Introduction

Coordinated adaptation to lactation is critical to the health and productivity of dairy cows during the transition period [1], and lipid metabolism plays a vital role in this adaptive process [2]. Excessive mobilization of adipose tissue to support milk synthesis in early lactation results in an increased risk of hyperketonemia and fatty liver syndrome [1,3], which can lead to productive and health losses [4]. One approach to minimizing the risk of periparturient problems is the manipulation of prepartum diets to meet the dietary needs of late gestation cows, concurrent with minimizing the risk of metabolic disorders [1].

Choline is used for phosphatidylcholine synthesis, a major phospholipid required for cell structure and function. The synthesis of phosphatidylcholines occurs by using absorbed choline or through trans-methylation of phosphatidylethanolamine by methyl donors such as s-adenosylmethionine [5]. Cows in early lactation have an inadequate supply of essential amino acids, including methionine, because dry matter intake (DMI) and the synthesis of microbial amino acids are limited in the first weeks postpartum, concurrent with the increased need for protein synthesis in colostrum and milk. Because intake and microbial synthesis of methionine may be limited in early lactation, it is possible that the availability of phosphatidylcholine is reduced, impairing lipid metabolism. Concentrations of phosphatidylcholine in plasma decrease from 14 days prepartum and seem to reach the nadir in the week of calving [6]. Collectively, the supply of choline may be limited mostly during the periparturient period in dairy cows, and supplementing diets with RPC during transition might benefit lactation performance [7].

In early lactation, dairy cows experience excessive lipid mobilization to support the synthesis of milk and milk fat [1]. Such adaptations to lactation can result in excessive accumulation of hepatic triacylglycerols because the rate of NEFA uptake by hepatocytes exceeds that of oxidation in the mitochondria and peroxisomes and exceeds their export as a very low-density lipoprotein. Excessive accumulation of hepatic triacylglycerol has been associated with changes in blood biochemical parameters and reduced performance in dairy cows [4,7,8]. Therefore, reducing hepatic triacylglycerol might benefit the metabolism and lactation of dairy cows. Zenobi et al. [9] demonstrated a dose-dependent effect of choline in reducing hepatic triacylglycerol in late gestation dry cows, and one of the mechanisms for reduced hepatic triacylglycerol by supplementing RPC is the increased secretion of nascent very low-density lipoproteins (VLDLs) in dairy cows [10]. As previously shown, as the intake of choline ion increased from 0 to 25.8 g/day, hepatic triacylglycerol decreased [9], thereby showing that RPC can reduce the risk of fatty liver in dairy cows [9,10]. Nevertheless, when fed to transition cows, RPC has shown mixed effects on hepatic triacylglycerol content postpartum [4,11]. In some cases, RPC reduced hepatic triacylglycerol [12,13,14], whereas in others, it had no effect [15,16].

We hypothesized that supplementing diets with RPC would improve the metabolism and lactation performance of early postpartum cows. For that, two experiments were conducted to determine whether supplementation with RPC, either only before calving or before and after calving, improves metabolism and lactation performance in dairy cows.

## 2. Materials and Methods

The University of California Davis Animal Care and Use Committee approved all procedures.

### 2.1. Cows and Housing

Two experiments were conducted. In experiment 1, 369 Holstein cows from farm 1 were used. Cows were housed in dry lots prepartum and free-stall barns postpartum, with four pens used per treatment per period. Individual dry lot pens were located in the same area, and individual free-stall pens were in the same barn. Pens were of identical design, size, and number of cows housed. Of the 369 cows initially enrolled, 7 were removed (4 cows died, and 3 cows were sold) before calving. In experiment 2, 578 primigravid Holstein cows from farm 2 were used. Cows were housed in 10 pens during the prepartum period, whereas postpartum cows were housed together, with primiparous cows separated from multiparous cows. Of the 578 cows initially enrolled, 5 were removed from data analyses because they were sold during the prepartum period.

### 2.2. Experimental Design and Treatments

Two experiments were conducted. In both, the experiments were randomized complete block designs with the pen as the experimental unit and the cows as the observational unit. A convenience sample size was used based on the availability of the number of pens and cows.

In experiment 1, the enrolment of cows lasted for 5 months, from November to March, coinciding with the winter and spring seasons. Weekly cohorts of cows were stratified by parity as nulliparous or parous cows and then randomly assigned to pens. Treatments were randomly assigned to each of the 8 pens to result in 4 prepartum pens per treatment, with 4 pens housing only nulliparous (2/treatment) and 4 housing only parous cows prepartum (2 per treatment). All cows were pregnant via artificial insemination, and they were assigned to pens at 253 ± 3 days of gestation. A total of 68 nulliparous and 115 parous cows were assigned to the control treatment, and 64 nulliparous and 115 parous cows were allocated to the RPC (ReaShure, Balchem Corp., Montvale, NJ, USA) treatment from 25 days prepartum to 80 days postpartum. Pre- and postpartum cows were locked once daily, in the afternoon, and RPC was fed individually as a top-dress. The RPC product contained 28.8% choline chloride (21.5% choline ion), and it was fed at 60 g/cow/day, providing approximately 12.9 g/cow/day of choline ion. Control cows received 60 g/cow/day of corn meal.

In experiment 2, the enrollment lasted for 3 months, from mid-December to the first week of March, and cows calved from January to the first week of April, coinciding with the winter and spring months. Pregnant primigravid cows were assigned to treatment groups according to a completely randomized design at 256 ± 3 days of gestation. Weekly, cows were stratified by age and randomly assigned to receive the control (n = 282; 5 pens) or RPC (n = 291; 5 pens) treatment. In experiment 2, cows received the treatments only during the prepartum period, and treatments were administered as described in experiment 1.

### 2.3. Diets, Diet Sampling, and Nutrient Analyses

In experiment 1, prepartum cows were fed once daily, and lactating cows were fed twice daily immediately after milking, whereas in experiment 2, cows were fed once daily, both pre- and postpartum. In both experiments, pen DMI were measured daily using feed management software (FeedWatch, Valley Ag Software, Tulare, CA, USA). The mixing wagons in each farm were fitted with a mixer scale indicator (GSE Scale Systems, Data Weighing Systems, Inc., Wood Dale, IL, USA), and the amounts of feed offered to and removed from individual pens were recorded daily. Individual ingredients were sampled twice weekly and analyzed for dry matter to adjust the quantities mixed.

In experiments 1 and 2, prepartum and lactation diets were formulated using the CPM-Dairy cattle ration analyzer (Cornell-Pen-Miner Ver. 3.0.7a, Ithaca, NY, USA). In experiment 1, diets were formulated to meet the requirements of prepartum cows weighing 670 kg and consuming 12 kg of dry matter in the last 3 weeks of gestation, and of lactating Holstein cows weighing 630 kg, consuming 23 kg of dry matter, and producing 45 kg/day of milk containing 35 g fat/kg of milk and 31 g of true protein/kg of milk in the first 80 days of lactation [17]. In experiment 2, diets were formulated to meet the requirements of prepartum nulliparous cows weighing 610 kg and consuming 10 kg of dry matter in the last 3 weeks of gestation, and of lactating primiparous Holstein cows weighing 560 kg, consuming 21 kg of dry matter, and producing 35 kg/day of milk containing 36 g of fat/kg of milk and 31 g of true protein/kg of milk in the first 80 days of lactation [17]. Details are displayed in Table 1 and Table 2.

Once weekly in each farm, 0.5 kg of complete diets and dietary ingredients were sampled and dried at 55 °C for 48 h and at 100 °C for 24 h in air-circulating ovens, and moisture losses were recorded. Samples dried at 100 °C were used to calculate DMI per pen. Samples dried at 55 °C were used for chemical analyses. Dried samples were ground to 2 mm in a Wiley Mill (Arthur H. Thomas Co., Chadds Ford, PA, USA) and then composited for each ingredient within the farm into 3 composite samples. Each composite sample was analyzed for dry matter at 105 °C, organic matter [18], acid detergent fiber and neutral detergent fiber [19], N using an N analyzer (FP-528 Nitrogen Determinator, LECO Corporation, St. Joseph, MI, USA), with crude protein calculated as N percentage multiplied by 6.25, and sulfuric acid-insoluble lignin. The ether extract was analyzed according to method 920.39 [18]. Minerals were analyzed in a commercial laboratory by inductively coupled plasma mass spectrometry (Dairyland Laboratory, Arcadia, WI, USA). Results of the chemical analyses of ingredients are in Table 3 and Table 4.

### 2.4. Lactation Performance and Body Condition Scoring

In experiment 1, cows were milked twice daily, and yields of milk and milk components were measured for individual cows once monthly by an official dairy herd improvement laboratory in Hanford, CA. Milk samples collected from the morning and evening milkings for the first 3 months postpartum were analyzed for somatic cells, fat, and true protein concentrations (Foss 303 Milk-O-Scan^®^; Foss Foods, Inc., Eden Prairie, MN, USA). Yields of milk corrected for 3.5% fat content and for energy, and the net energy content of milk were calculated as follows: 3.5% fat-corrected milk, kg/day = 0.4324 × milk kg + (16.218 × milk fat kg); energy-corrected milk, kg/day = [(0.3246 × milk yield) + (12.86 × fat yield) + (7.04 × protein yield)]; net energy for lactation, Mcal/kg = (0.0929 × fat %) + (0.0563 × protein %) + (0.0395 × lactose %), according to NRC [17].

In experiment 2, cows were milked three times daily, and yields of milk were measured for individual cows daily during the first 80 days postpartum. Daily milk yield was averaged into weekly means for statistical analysis.

In experiment 1, the body condition of all cows was scored at enrollment and at 1, 30, 60, and 90 days postpartum, whereas in experiment 2, it was evaluated at enrollment and at calving, using a scale from 1 to 5, according to Ferguson et al. [20].

### 2.5. Blood Sampling and Analyses of Blood Metabolites

Blood was sampled by puncture of the coccygeal vessels using K2 EDTA tubes (Becton Dickinson, Franklin Lakes, NJ, USA). Samples were placed in ice, centrifuged at 1500× *g* for 15 min, and plasma was frozen at −25 °C until assays.

In experiment 1, plasma concentrations of glucose and NEFAs were analyzed in a subset of 32 primiparous (control = 15; RPC = 17) and 48 multiparous (control = 24; RPC = 24) cows, representing all 8 pens at calving (1 ± 1 day postpartum) and at 14 ± 1 and 21 ± 1 days postpartum. The concentration of total choline in plasma was analyzed in a subset of 46 cows, 26 primiparous (control = 14; RPC = 12) and 20 multiparous (control = 10; RPC = 10) cows, representing all 8 pens (4 to 8 cows/pen), on days 1 and 14 postpartum. Blood also was sampled from all cows at 0 and 14 days postpartum and analyzed for concentrations of BHB. Plasma samples collected on days 0 and 14 postpartum from 26 primiparous (control = 14; RPC = 12) and 20 multiparous (control = 10; RPC = 10) cows representing all 8 pens (4 to 8 cows/pen) were analyzed for choline concentrations. These were the same cows that had liver samples collected for analyses of triacylglycerol and glycogen contents.

In experiment 2, concentrations of glucose and NEFAs were measured in plasma sampled from a subset of 22 control and 25 RPC cows, representing the ten prepartum pens, at 0, 14, and 21 days postpartum. Plasma was also analyzed on days 1 and 14 postpartum for concentrations of choline. Additional blood was sampled from all cows at calving and 14 days postpartum for analysis of BHB in plasma.

All samples were analyzed in duplicate and samples with CV > 10% were re-analyzed. Glucose was analyzed by direct measurement using the YSI Model 2700 SELECT Biochemistry Analyzer (YSI Co., Inc., Yellow Springs, OH, USA), and the mean CV was 3.8%. The concentration of NEFAs was analyzed using a commercial kit (NEFA C, Wako Chemicals USA, Inc., Richmond, VA, USA) with the method described by Johnson and Peters (1993) [21], and the mean CV for the assays was 6.6%. Plasma BHB was analyzed using a commercial kit (Randox Laboratories Ltd., Crumlin, UK) and the mean CV for the assays was 7.7%. Cows were considered to have hyperketonemia when BHB > 1.20 mmol/L. The concentration of choline in plasma was determined by liquid chromatography with tandem mass spectrometry at the University of California Davis metabolomics core, according to Holm et al. [22].

### 2.6. Liver Tissue Collection and Analysis

In experiment 1, the liver samples were collected from the same subset of cows whose plasma was analyzed for choline, 26 primiparous (control = 14; RPC = 12) and 20 multiparous (control = 10; RPC = 10) cows, representing all 8 pens (4 to 8 cows/pen), between days 7 and 10 postpartum (mean ± SD = 9.0 ± 1.3 and median = 9.0). After a thorough cleansing of the 11th intercostal space on the right side, 15 mL of a 2% lidocaine HCl solution was injected subcutaneously and intramuscularly. The skin was incised, and the liver biopsy needle was introduced through the intercostal muscle layer until it reached the liver. Approximately 500 mg of the liver was sampled per cow. The tissue was dried, rinsed with sterile saline, and placed in sterile vials that were snap-frozen in liquid N and transferred to a −80 °C freezer for later analyses.

Triplicates of ~50 mg of liver sample were placed in 2 mL of ice-cold PBS and homogenized with a tissue homogenizer (Fisher PowerGen 35; Fisher Scientific, Hampton, NH, USA). Free glucose was analyzed by direct measurement as described previously for plasma. Immediately after, 1.0 mL of homogenate was mixed with 500 µL of a 4 M HCl solution and boiled for two h. After boiling, samples were centrifuged at 2000× *g* for 15 min. A total of 700 µL of the supernatant were neutralized with 375 µL of Tris buffer (2 M, pH = 8.8) and 250 µL of a 2 M NaOH solution and then analyzed for glucose concentration. Glycogen content in tissue was calculated after subtracting the free glucose from the total glucose concentration.

The concentrations of triacylglycerol in the liver samples were determined using a commercial kit (Triglycerides-L-Type TG H, Wako Chemicals USA, Inc., Richmond, VA, USA). The liver dry matter was analyzed after drying 300 µL of the initially homogenized liver tissue in PBS at 100 °C for 6 h. Concentrations of triacylglycerol and glycogen were expressed as % of the liver on a tissue wet basis or tissue dry matter basis.

### 2.7. Statistical Analyses

Data from experiments 1 and 2 were analyzed separately, and within an experiment, data were analyzed separately for the pre- and postpartum periods. The cow was the unit of measure, and the pen was the experimental unit. The random effect of the pen nested within treatment was the error term with which to test the effect of treatment in all statistical models, except for the analysis of DMI, in which the error term was the residual variance.

Continuous variables were analyzed by ANOVA using the MIXED procedure of SAS (SAS/STAT ver. 9.4, SAS Institute Inc., Cary, NC, USA). The statistical model used is as follows:

Y_ijkl_ = μ + T_i_ + W_j_ + T_i_ × W_j_ + *P*(*T*)*_ki_* + *C*(*P* × *T*)*_lki_* + *e_ijkl_*

where Y_ijkl_ is the dependent variable, μ is the overall mean, T_i_ is the fixed effect of treatment (i = control vs. RPC), W_j_ is the fixed effect of time (day, week, or month; j = 1 to n), T_i_ × W_j_ is the fixed effect of interaction between T_i_ and W_j_, *P*(*T*)*_ik_* is the random effect of the pen (k = 1 to n) nested within treatment, *C*(*P* × *T*)_lki_ is the random effect of the cow (l = 1 to n) nested within the pen by treatment, and *e_ijkl_* is the random residual. In experiment 1, the fixed effect of parity (primiparous vs. multiparous) was also included in the statistical models. Time was the term in the REPEATED statement, the subject was the cow nested within treatment by the pen, and the variance–covariance structure was selected based on model convergence and fit statistics was assessed using the smallest corrected Akaike’s information criterion. In all mixed models, the Kenward–Roger method was used to approximate the denominator degrees of freedom to compute the *F* tests. The distribution of residuals and homogeneity of variance were evaluated after the model fit. Concentrations of BHB and NEFAs in plasma and the liver content of triacylglycerols were transformed to the natural logarithm to achieve normality of residuals and homogeneity of variance, and the least squares means and respective standard errors of the means were back-transformed for data presentation according to Jørgensen and Pedersen [23].

For DMI, the daily value of dry matter consumed by the pen was divided by the number of cows in the pen. A single mean per pen was obtained for the pre-and postpartum periods for statistical analyses. The statistical model used is as follows:

Y_i_ = μ + T_i_ + *e_i_*

where Y_i_ is the dependent variable, μ is the overall mean, T_i_ is the fixed effect of treatment (i = control vs. RPC), and *e_i_* is the random residual. In experiment 1, the effect of parity was also included in the statistical models.

The prevalence of subclinical ketosis, the incidence of subclinical ketosis, and the prevalence of hepatic lipidosis were analyzed by logistic regression using the GLIMMIX procedure of SAS with binary distribution and a logit link function. The statistical model used is as follows:

Ln [(P_1_)/(P_0_)] = β_0_ + β_1_·T_i_ + β_2_·*P*(*T*)*_ji_*

where Ln is the natural log, P_1_ is the probability of the event = 1, P_0_ is the probability of the event = 0, β_0_ is the intercept of the logistic regression, β_1_·T_i_ is the beta coefficient for the fixed effect of treatment (i = control vs. RPC), and *P*(*T*)*_ji_* is the random effect of the pen (j = 1 to n) nested within treatment. The adjusted odds ratios and the respective 95% confidence intervals (CIs) were calculated.

Regression analysis was performed to establish the relationship between the plasma concentration of choline and liver triacylglycerol. The Log_10_ of liver triacylglycerol was used, and regression analyses were performed with either each treatment separately or with both combined using the PROC REG of SAS.

Treatment differences with *P* < 0.05 were considered significant, and 0.05 < *P* < 0.10 was designated as a tendency.

## 3. Results

The length of the feeding of prepartum diets in experiment 1 did not differ (*P* = 0.37) between treatments, and the mean (±standard deviation) and median days were 25.0 ± 5.9 and 25 days for the control treatment and 24.6 ± 6.7 and 25 days for the RPC treatment. In experiment 2, the days of treatment did not differ (*P* = 0.43), and the mean and median days were, respectively, 22.5 ± 5.6 and 22 days for the control treatment and 22.0 ± 5.7 and 22 days for the RPC treatment. In both experiments, the minimum and maximum number of days that a cow spent receiving the treatment diets were 11 and 47 days, respectively.

### 3.1. Productive Performance

In experiment 1, prepartum DMI tended (*P* = 0.10) to be greater for cows fed RPC compared with control cows (Table 5). Similarly, postpartum DMI tended (*P* = 0.06) to be greater for the RPC compared with the control treatment. Supplementing RPC before and after calving tended (*P* = 0.10) to increase milk yield by 1.4 kg/d in the first 80 days postpartum. Similarly, RPC increased (*P* = 0.05) yields of 3.5% fat-corrected milk and energy-corrected milk compared with the control. The contents of fat and true protein in milk did not differ between treatments, but supplementing with RPC increased (*P* = 0.04) yield of fat by 92 g/d and tended (*P =* 0.08) to increase the yield of true protein by 44 g/d compared with the control. Although the net energy content in milk did not differ with treatment, cows fed RPC secreted more (*P* = 0.05) Mcal as milk energy than those fed the control treatment. The somatic cell count was low in the experiment, and the somatic cell score did not differ between treatments, with a mean of 1.64. Treatment did not affect the changes in body condition pre- or postpartum, but cows fed RPC had greater (*P* = 0.02) mean body condition postpartum than control cows (Table 5).

In experiment 2, feeding with RPC tended (*P* = 0.10) to increase DMI prepartum (control = 11.3 ± 0.2 vs. RPC = 11.8 ± 0.2 kg/day). Milk yield in primiparous cows fed RPC prepartum tended (*P* = 0.10) to increase (control = 26.4 ± 0.4 vs. RPC = 27.4 ± 0.4 kg/day) in the first 80 days postpartum compared with the control. The body condition of the cows on the day after calving tended (*P* = 0.10) to be greater (control = 3.72 ± 0.02 vs. RPC = 3.69 ± 0.02) for control than for RPC cows. Control cows tended (*P* = 0.09) to have a smaller change in body condition between enrollment and calving (control = −0.07 ± 0.02 vs. RPC = −0.13 ± 0.02) compared to those fed RPC.

### 3.2. Concentrations of Choline, Glucose, and Nonesterified Fatty Acids in Plasma

**Figure 1:**
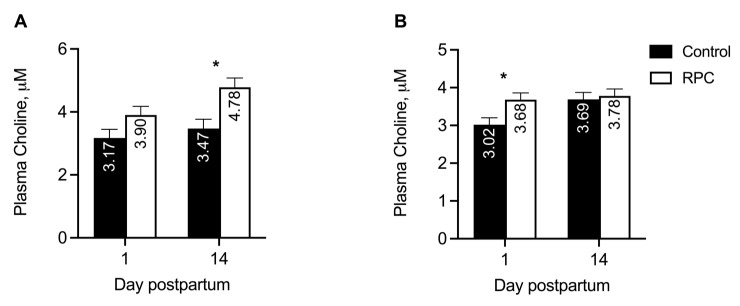
Effects of feeding rumen-protected choline (RPC) on plasma concentrations of choline on days 1 and 14 postpartum in experiments 1 (**A**) and 2 (**B**). Treatments were from 25 days before to 80 days after calving (experiment 1) or during the last 22 days of gestation (experiment 2). Error bars represent the standard errors of the means, and the pooled values were 0.28 and 0.18 in experiments 1 and 2, respectively. Panel A: effects of treatment (*P =* 0.08) and interaction between treatment and day (*P =* 0.007). Panel B: effects of treatment *(P =* 0.12) and interaction between treatment and day (*P =* 0.04). * Within day, effect of treatment (*P <* 0.05).

An interaction (*P* = 0.007) between treatment and day postpartum was observed for plasma choline concentrations in experiment 1 because feeding with RPC did not increase (*P* = 0.16) choline in plasma of cows on day 1 but increased (*P* = 0.04) on day 14 (Figure 1A). The concentrations of choline in the plasma had means of 3.32 ± 0.27 and 4.34 ± 0.28 µM in control and RPC cows, respectively, in experiment 1. In experiment 2, the interaction (*P* = 0.04) between treatment and day also affected plasma choline concentrations because feeding with RPC increased (*P* = 0.02) choline on day 1 postpartum but not on day 14 (*P* = 0.72; Figure 1B). The concentrations of choline in the plasma had means of 3.35 ± 0.16 and 13.73 ± 0.15 µM in control and RPC cows, respectively.

**Figure 2:**
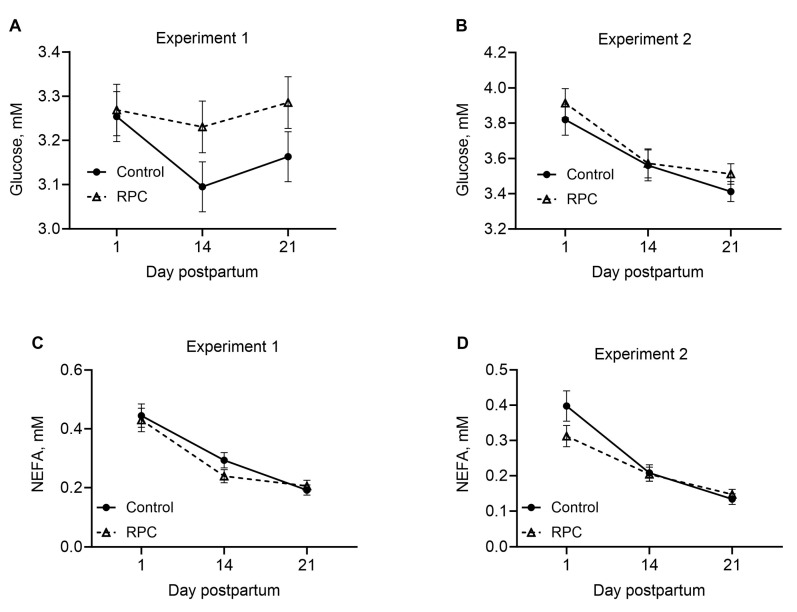
Effects of feeding rumen-protected choline (RPC) on concentrations of glucose (**A**) and nonesterified fatty acids (NEFAs, (**C**)) in plasma in experiment 1 and glucose (**B**) and NEFAs (**D**) in experiment 2. Treatments were from 25 days before to 80 days after calving (experiment 1) or during the last 22 days of gestation (experiment 2). Error bars represent standard errors of means, and the pooled values were 0.06 and 0.03 for glucose and NEFAs in experiment 1 and 0.09 and 0.02 for glucose and NEFAs in experiment 2. Panel (**A**): effects of treatment (*P =* 0.17) and interaction between treatment and day postpartum (*P =* 0.43). Panel (**B**): effects of treatment (*P =* 0.51) and interaction between treatment and day postpartum (*P =* 0.21). Panel (**C**): effects of treatment (*P =* 0.50) and interaction between treatment and day postpartum (*P =* 0.67). Panel (**D**): effects of treatment (*P =* 0.59) and interaction between treatment and day postpartum (*P =* 0.27).

In experiment 1, treatment or the interaction between treatment and day did not affect the concentrations of glucose or NEFAs in the plasma of postpartum cows (Figure 2A,B). Similarly, in experiment 2, neither treatment nor the interaction between treatment and day affected the concentrations of glucose and NEFAs in the plasma (Figure 2C,D).

### 3.3. Concentration of BHB in Plasma, Hyperketonemia, and Hepatic Tissue Composition

**Figure 3:**
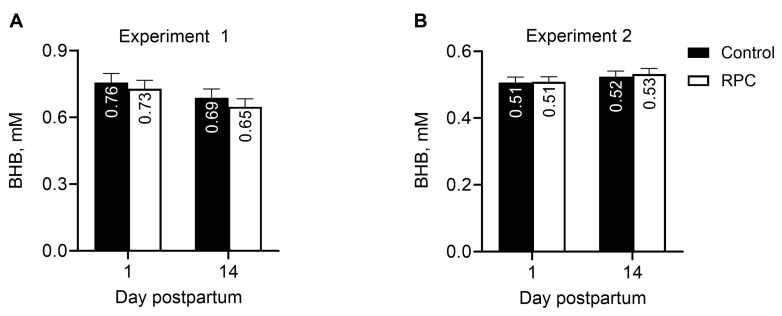
Effect of feeding rumen-protected choline (RPC) on concentrations of beta-hydroxybutyrate (BHB) in plasma of dairy cows in experiments 1 (**A**) and 2 (**B**). Treatments were from 25 days before to 80 days after calving (experiment 1) or during the last 22 days of gestation (experiment 2). Error bars represent standard errors of means, and the pooled values were 0.034 and 0.016 in experiments 1 and 2, respectively. Panel (**A**): effects of treatment (*P =* 0.50) and interaction between treatment and day postpartum (*P =* 0.78). Panel (**B**): effects of treatment (*P =* 0.79) and interaction between treatment and day postpartum (*P =* 0.87).

Treatment did not affect the concentrations of BHB in experiment 1, and no interactions between treatment and day or treatment and parity group were observed (Figure 3A). Similarly, treatment did not affect the concentrations of BHB in experiment 2 (Figure 3B).

**Figure 4:**
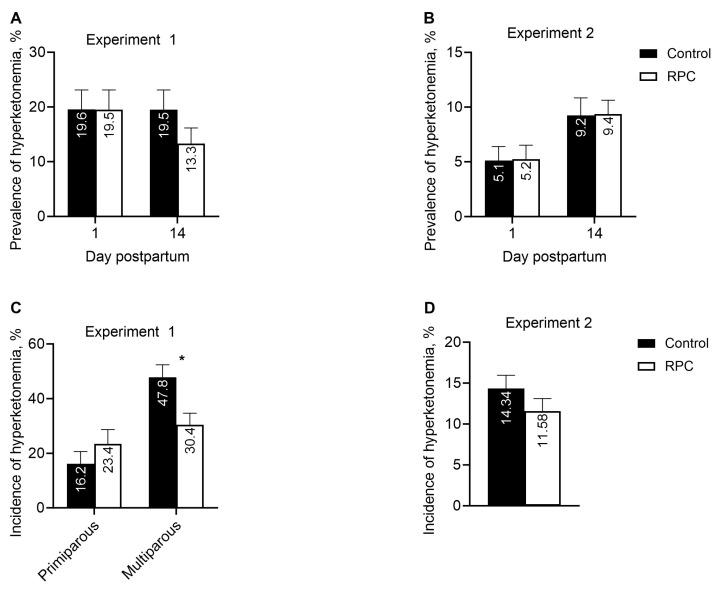
Effect of feeding rumen-protected choline (RPC) on prevalence of hyperketonemia based on plasma BHB > 1.20 mmol/L in experiments 1 (**A**) and 2 (**B**) and on incidence of hyperketonemia in experiments 1 (**C**) and 2 (**D**). Treatments were from 25 days before to 80 days after calving (experiment 1) or during the last 22 days of gestation (experiment 2). Error bars represent standard errors of means, and the pooled values were 3.37 and 1.43 for prevalence of hyperketonemia in experiments 1 and 2, respectively, and 4.63 and 1.57 for incidence of hyperketonemia in experiments 1 and 2, respectively. Panel (**A**): effects of treatment (*P =* 0.45) and interaction between treatment and day postpartum (*P =* 0.17). Panel (**B**): effects of treatment (*P =* 0.94) and interaction between treatment and day postpartum (*P =* 0.98). Panel (**C**): effects of treatment (*P =* 0.62) and interaction between treatment and parity (*P =* 0.08). Panel (**D**): effect of treatment (*P =* 0.25). * Within parity group, effect of treatment (*P =* 0.05).

Treatment did not affect the daily prevalence of hyperketonemia in either experiment (Figure 4A,C), and the prevalence was greater in experiment 1 than 2. Nevertheless, feeding with RPC reduced (*P* = 0.05) the incidence of hyperketonemia within multiparous cows in experiment 1 (Figure 4B) but not in primiparous cows in both experiments.

**Figure 5:**
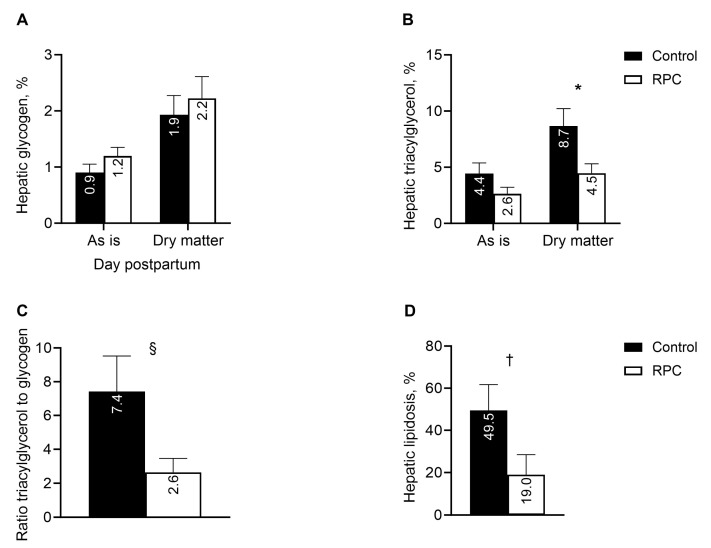
Effect of feeding rumen-protected choline (RPC) on hepatic composition in experiment 1. Treatments were from 25 days before to 80 days after calving. Hepatic glycogen (**A**), triacylglycerol (**B**), ratio of triacylglycerol to glycogen (**C**), and hepatic lipidosis based on triacylglycerol concentration >5% on a tissue as is basis (**D**). Error bars represent standard errors of means, and pooled values for as is and dry matter bases were, respectively, 0.15 and 0.38 (**A**), 0.77 and 1.20 (**B**), 1.9 (**C**), and 10.8 (**D**). * Effect of treatment (*P =* 0.05); ^§^ Effect of treatment (*P =* 0.07); ^†^ Effect of treatment (*P =* 0.10).

Liver tissue was sampled from cows only in experiment 1 at 9.0 ± 1.3 days postpartum (± SD). Feeding with RPC did not affect the concentration of glycogen in the hepatic tissue expressed on tissue as is or dry matter bases (Figure 5A); however, feeding with RPC reduced (*P* = 0.05) the concentration of triacylglycerol on a liver dry matter basis (Figure 5B). The concentrations of triacylglycerols on liver as is and liver dry matter bases in cows fed RPC represented, respectively, 59.3 and 51.6% of those in the control cows. The reduction in triacylglycerols in the liver tissue of cows fed RPC resulted in a tendency (*P* = 0.07) to reduce the ratio of triacylglycerol to glycogen and a tendency (*P* = 0.10) to reduce the risk of hepatic lipidosis compared with control cows (Figure 5C,D).

**Figure 6:**
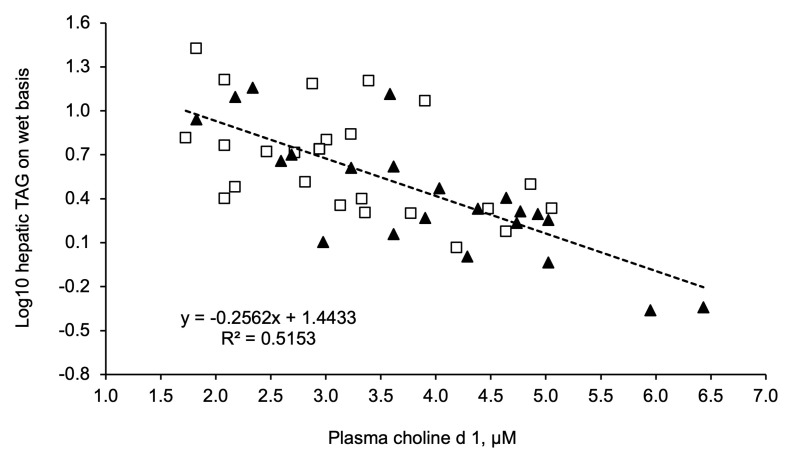
Relationship between plasma choline concentration (µmol/L) on day 1 postpartum and hepatic triacylglycerol (Log_10_ of wet weight mg/100 mg) on day 9 postpartum in cows in experiment 1 according to dietary treatment, control (□) and RPC (▲). In both treatments, a linear (*P <* 0.0001) relationship was observed. Concentration of hepatic triacylglycerol (Log_10_) = 1.4433 − 0.2562 × plasma choline (µmol/L); R^2^ = 0.52.

Interestingly, a linear (*P* < 0.001) relationship between plasma choline concentration and hepatic triacylglycerol content was observed in experiment 1. As the concentration of choline in the plasma on day 1 postpartum increased, that of triacylglycerol in the hepatic tissue on day 9 postpartum decreased (Figure 6). For each 1 µmol/L increase in choline concentration in the plasma, the content of triacylglycerol in the hepatic tissue decreased by 0.256 logarithms on the base 10 (R^2^ = 0.52).

## 4. Discussion

The present experiment, conducted on commercial dairy farms, employed a pen-based experimental design to investigate the effects of feeding rumen-protected choline (RPC) on production performance and metabolism in dairy cows. The findings showed that feeding with RPC improved yields of milk and energy-corrected milk, and metabolism, and the benefits were greater in experiment 1, when RPC was fed both before and after calving. Although comparisons between the two experiments herein are not appropriate, the present data show that supplementing with RPC only prepartum in nulliparous cows results in a limited milk production response. Nevertheless, the practical implication of the current experiment corroborates that feeding with RPC during the transition period reduces the risk of hepatic lipidosis and improves yields of milk and milk components. Supplementing diets of transition cows with RPC improved yields of fat-corrected milk and energy-corrected milk in experiment 1 and tended to improve the yields of milk in experiment 2. In both experiments, diets were formulated with supplemental undegraded protein sources and rumen-protected methionine to minimize potential insufficiency in metabolizable methionine. The response to choline increases when diets are inadequate in methionine [4], presumably because methionine can be used to synthesize S-adenosyl-methionine that serves as a methyl donor for the synthesis of phosphatidylcholine [5]. Recent work showed that lactation response to choline is amplified when postpartum diets are limited in metabolizable methionine [4]. Because pre- and postpartum diets supplied at least 20 g of metabolizable methionine per kg of metabolizable protein, it is suggested that improvements in the performance of dairy cows were not expected to be exacerbated by dietary methionine inadequacy [4,24].

Supplementing transition cows with RPC tended to increase DMI prepartum in both experiments. Furthermore, feeding with RPC before and after calving also tended to increase DMI postpartum in experiment 1. In general, supplementing with RPC during the transition period results in small increments in DMI observed both pre- and postpartum [4]. Nevertheless, the changes are rather small, in the magnitude that ranges from 0.2 to 0.5 kg/d when cows are fed 12.9 g/d of supplemental choline ion as RPC [4], which probably explains why most individual experiments report no effects of RPC on intake in dairy cows [16,25,26]. Nevertheless, increases in intake with RPC have been observed in individual experiments [27], corroborating the present findings. The mechanisms by which RPC might influence DMI remain unknown, but an indirect effect mediated by improved periparturient health is a possibility. Cows fed RPC tended to have less risk of retained fetal membranes and mastitis [4], which might explain the observed tendency for greater intake in the present experiment. Also, RPC supports improved hepatic health with reduced concentrations of hepatic triacylglycerol, which was observed herein in experiment 1. Increased concentration of hepatic triacylglycerol are linked with less DMI [11], and lipidosis of the hepatic tissue can affect hepatic health, which might influence appetite in dairy cows. The improvements in DMI postpartum in experiment 1 help to partially explain the increases in energy-corrected milk and the greater mean BCS in cows fed RPC compared with the controls.

Several experiments have evaluated the impact of RPC during the transition on lactation performance. A review article [28] and a systematic review of the literature and meta-analysis [4] clearly documented the positive effects of supplementing diets with RPC during the transition period on yields of milk and milk components. Baldi and Pinotti [28] showed that the increase in milk yield for cows fed diets supplemented with RPC relative to the same diet without supplemental RPC was superior by 6% in 8 of the 12 experiments compared. Arshad et al. [4] showed that supplementing diets with RPC starting prepartum increased energy-corrected milk yield by 2.18 kg/d, with moderate heterogeneity. The authors demonstrated that variability in response to RPC was affected by the amount of choline ion supplemented and the supply of metabolizable methionine in the postpartum diets [4]. Even when RPC is supplemented only postpartum, improvements in milk yield seem to depend on the amount of choline ion fed [25].

Increased yields of milk components from feeding RPC generally result from increased milk production, with some effects on milk fat content but little or no effect on milk protein content. In experiment 1, treatment with RPC did not influence concentrations of fat, protein, or the energy content of milk. Because choline is used for phospholipid synthesis, it has been suggested that its supplementation may facilitate lipid absorption and transport [9]. However, detailed work on blood lipids revealed that RPC did not alter different lipid fractions in the plasma of lactating cows [29]. In early lactation, plasma NEFAs supply a large portion of the fatty acids secreted by the mammary gland in dairy cows [30], and this contribution can increase if weight loss postpartum is exacerbated. Limited evidence exists to suggest that supplementing diets with RPC affects body fat mobilization in transition cows [4]. When late-pregnant dry cows are subjected to controlled feed restriction [9,10], blood NEFA concentrations do not differ between control and RPC-supplemented cows, thus suggesting that supplementing choline does not have direct effects on adipose tissue mobilization. Indeed, treatment in the present experiment did not affect plasma NEFAs, and no differences in the content of fat in milk between control and RPC cows were observed in experiment 1.

The inhibition of endogenous choline synthesis from methionine in lactating dairy cows reduced the yields of milk and fat-corrected milk when cows received methionine infusion but not when they received choline [31]. The latter finding confirms that methionine is an important methyl donor in lactating dairy cows and presumably is used for the synthesis of phosphatidylcholines. Nevertheless, multiparous cows fed diets formulated to be inadequate in methionine had increased milk production when supplemented with RPC but not when supplemented with rumen-protected methionine or betaine [24]. The same response to RPC was not observed in primiparous cows, likely because milk production was low, ~27.5 kg/day [24]. Although the response to RPC is influenced by the postpartum supply of methionine, increments in yields of milk and energy-corrected milk were observed even when multiparous cows were fed diets with 26.2 g of metabolizable methionine per kg of metabolizable protein [4]. It is possible that improvements in lactation performance in response to choline supplementation may be attributed to a methyl donor-sparing effect but also to a direct effect of choline in supplying a substrate for phospholipid synthesis. Moreover, supplementation with RPC might alleviate the deposition of triacylglycerol in the hepatic tissue [9,10] and reduce the risk of some periparturient diseases [4], which might favor increased DMI and productive performance.

Supplementing pre- and postpartum diets with RPC in experiment 1 reduced the risk of hyperketonemia in multiparous cows; however, the same response was not observed in primiparous cows in experiments 1 and 2. The prevalence of hyperketonemia in the first 14 days postpartum was low in primiparous cows, particularly in experiment 2, which might explain the inability of RPC to have any effect. Primiparous cows seem to be less prone to an accumulation of hepatic triacylglycerol compared to multiparous cows [32], perhaps because they produce less than multiparous cows in early lactation, which would have less impact on body fat mobilization and subsequent ketogenesis. Feeding with RPC increased the concentration of choline in the plasma of cows in both experiments, although the response was time-dependent and observed only on day 14 postpartum in experiment 1 and on day 1 in experiment 2. The increases in plasma choline indicate that the choline ion present in the RPC product supplemented was likely absorbed and delivered to the systemic circulation [33], which could affect the supply of choline and phosphatidylcholines to numerous tissues, including the liver and mammary gland. The concentration of triacylglycerol in the hepatic tissue was less in cows supplemented with RPC compared with those fed the control diet. Also, RPC tended to reduce the ratio of triacylglycerol to glycogen in the hepatic tissue as well as reducing the risk of hepatic lipidosis. Arshad et al. [10] showed that supplementing RPC to diets of feed-restricted late gestation cows alleviated the accumulation of triacylglycerol in the hepatic tissue, in part by increasing hepatic secretion of nascent VLDLs. The same group [10,11] proposed numerous mechanisms by which supplementing choline ions as RPC might facilitate the disposal of triacylglycerol from the hepatic tissue in dairy cows. Changes in the hepatic tissue composition of dairy cows during the postpartum period because of supplemental RPC have been observed by others [12,13,14], although responses have not been consistent [16,26]. A previous experiment [9] showed that RPC’s ability to reduce hepatic triacylglycerol was dependent on the dose, and the response was linear up to 25.9 g/day of choline ion fed in a rumen-protected form [9].

It is interesting to note that the content of hepatic triacylglycerol was linearly and negatively related to the concentrations of choline in plasma in the present experiment. These findings suggest that supplementing choline to increase the postabsorptive supply to the liver [33] might minimize the risk of hepatic lipidosis. Using the feed-restricted late-pregnant dry cow model [9], a previous experiment showed that as the amount of choline ion supplemented increased, the triacylglycerol content of the hepatic tissue decreased. Arshad et al. [10] showed that choline stimulated hepatic secretion of VLDLs, and in vitro experiments have demonstrated that supplementing choline as choline chloride to bovine hepatocytes stimulated secretion of VLDLs [34], which would support increased export of hepatic fatty acids as triacylglycerols [10,11,15]. Collectively, these data suggest that the mechanisms by which RPC minimizes hepatic infiltration of triacylglycerol might be mediated by changes in the hepatic metabolism of fatty acids that, among other features, result in increased export by the liver as VLDLs [11].

A limitation of the present study is the fact that only milk production was measured in experiment 2, without information on milk composition. A second limitation is that only two treatments were implemented in each experiment, with a single amount of RPC used, which precludes further implications of the findings and limits comparisons between the two experiments. Nevertheless, a strength of the present study is the fact that these on-farm multi-pen experiments corroborate the findings of university-led experiments with individual cow feeding.

## 5. Conclusions

Supplementing transition dairy cows with RPC from 25 days before to 80 days after calving tended to improve pre- and postpartum DMI, which favored productive performance. Cows fed RPC before and after calving had increased yields of 3.5% fat-corrected milk, energy-corrected milk, fat, and true protein. When fed only prepartum to primigravid cows, RPC tended to increase DMI prepartum and milk yield in the first 80 days postpartum. Supplementing diets fed to transition cows with RPC before and after calving increased choline concentrations in plasma and reduced the risk of hepatic lipidosis. A linear negative relationship between plasma choline and hepatic triacylglycerol content was observed, and cows with smaller concentrations of choline in the plasma were those with increased hepatic triacylglycerol content. The results reported herein corroborate findings from experiments with cows individually fed RPC and provide additional support of the importance of choline nutrition during the transition period in dairy cows to improve metabolism and production performance in early lactation. An implication of the present experiments is that response to RPC in nulliparous cows, when fed only prepartum, seems less than that typically observed when RPC is fed during the entire transition period.

## Figures and Tables

**Table 1:** Ingredient composition of diets and grain mixtures.

	Diets ^1^			
	Experiment 1		Experiment 2	
Item	Prepartum	Postpartum	Prepartum	Postpartum
	g/kg, dry matter basis			
Corn silage	348	195	444	322
Alfalfa hay, dry cow	218	---	222	---
Alfalfa hay, lactating cow	---	133	---	136
Alfalfa silage	---	62	---	---
Sudan grass silage	---	44	---	---
Citrus pulp	217	89	---	59
Grain mixture	217	477	334	483
Composition of grain mixtures				
Steam-flaked corn, 390 g/L	---	550	269	422
Corn distillers’ grains	784	130	201	41
Almond hulls	---	---	332	106
Canola meal, solvent extract	---	---	---	70
Soybean meal, solvent extract	---	---	---	70
Heat-treated soybean meal ^2^	---	---	132	70
Whole cottonseed	---	130	---	105
Ca salts of palm fatty acids ^3^	---	22	---	---
Prepartum mineral supplement ^4^	124	---	66	---
Protein–mineral supplement ^5^	---	137	---	106
Monensin–Se supplement ^6^	92	31	---	---
Beef tallow	---	---	---	10

^1^ Prepartum diets were fed during the last 25 and 22 days of gestation for experiments 1 and 2, respectively; postpartum diets were fed during the first 80 days of lactation. ^2^ Amino Plus (Ag Processing, Inc., Emmetsburg, IA, USA). ^3^ Ener GII (Virtus Nutrition, LLC, Corcoran, CA, USA). ^4^ Experiment 1: contained (per kg dry matter basis) 321 g of anhydrous calcium chloride, 321 g of anhydrous magnesium sulfate, 49 g of magnesium oxide, 44 g of Zinpro 4-Plex (mixture of zinc methionine, manganese methionine, copper lysine, and cobalt glucoheptonate; Zinpro Co., Eden Prairie, MN, USA), 173 g of Diamond V XP (culture of *Saccharomyces cerevisiae*; Diamond V Mills, Cedar Rapids, IA, USA), 12.6 g of a mixture of iodine and vitamins, and 79.4 g of corn germ meal. Supplement contained (per kg dry matter basis): 136 g Ca, 1 g P, 82 g Mg, 2 g K, 81 g S, 0.4 g Na, 237 g Cl, 1240 mg Zn, 430 mg Cu, 690 mg Mn, 86 mg Co, 5.5 mg I, 210,000 IU of vitamin A, 40,000 IU of vitamin D, and 12,000 IU of vitamin E. Experiment 2: contained (per kg dry matter basis) 473 g of corn meal, 132 g of monocalcium phosphate, 66 g of magnesium oxide, 66 g of magnesium sulfate anhydrous, 50 g of Zinpro 4-Plex, 173 g of Diamond V XPC, and 40 g of a mixture of iodine, selenium, vitamins, and Rumensin 80 (176 mg of monensin per kg; Elanco Animal Health, Greenfield, IN, USA). Supplement contained (per kg dry matter basis): 26 g Ca, 33 g P, 44 g Mg, 3 g K, 13 g S, 0.8 g Na, 1 g Cl, 1454 mg Zn, 493 mg Cu, 794 mg Mn, 99 mg Co, 23 mg I, 15.6 mg of Se, 330,000 IU of vitamin A, 88,000 IU of vitamin D, 13,500 IU of vitamin E, and 1,500 mg of sodium monensin. ^5^ Experiment 1: contained (per kg dry matter basis) 238 g Pro-Lak (blend of marine and animal by-products; H. J. Baker & Bro., Inc., Stamford, CT, USA), 518 g Amino Plus (heat-treated soybean meal; Ag Processing, Inc., Emmetsburg, IA, USA), 3.6 g Alimet (hydroxy methyl-thio butanoic acid; Novus International, Inc., St. Louis, MO, USA), 3.6 g Smartamine M (polymer-protected D, L-methionine, Adisseo Inc., Alpharetta, GA, USA), 28.5 g Diamond V XP (culture of *Saccharomyces cerevisiae*; Diamond V Mills, Cedar Rapids, IA, USA), 131 g sodium bicarbonate, 26 g monocalcium phosphate, 40 g magnesium oxide, 7.1 g Zinpro 4-Plex (mixture of Zn methionine, Mn methionine, Cu lysine, and Co glucoheptonate; Zinpro Co., Eden Prairie, MN, USA), 0.24 g Zn sulfate, and 4 g of a mixture of iodine and vitamins. Supplement contained (per kg dry matter basis): 440 g crude protein, 25 g Ca, 17 g P, 30 g Mg, 32 g K, 8 g S, 37 g Na, 2 g Cl, 515 mg Zn, 116 mg Cu, 125 mg Mn, 12 mg Co, 9.5 mg I, 50,000 IU of vitamin A, 11,000 IU of vitamin D, and 500 IU of vitamin E. Experiment 2: contained (dry matter basis) 329 g Pro-Lak, 207 g porcine blood meal, 3.6 g Alimet, 6 g Smartamine M, 20 g Diamond V XPC (culture of *Saccharomyces cerevisiae* concentrated formula; Diamond V Mills, Cedar Rapids, IA, USA), 168 g sodium bicarbonate, 30 g monocalcium phosphate, 42 g magnesium oxide, 90 g magnesium sulfate heptahydrate, 60 g calcium carbonate, 30 g potassium carbonate, 10 g Zinpro 4-Plex, 0.6 g zinc sulfate, and 3.8 g of a mixture of iodine, selenium, vitamins, and Rumensin 80. Supplement contained (per kg dry matter basis): 439 g crude protein, 35 g Ca, 13 g P, 34 g Mg, 20 g K, 19 g S, 51 g Na, 2 g Cl, 535 mg Zn, 95 mg Cu, 151 mg Mn, 19 mg Co, 12.6 mg I, 5.6 mg Se, 68,000 IU of vitamin A, 15,000 IU of vitamin D, 800 IU of vitamin E, and 310 mg of sodium monensin. ^6^ Contains (per kg dry matter basis) 1350 mg of sodium monensin and 19 mg of Se from sodium selenite with corn germ meal as carrier.

**Table 2:** Nutrient composition of diets (mean ± SD) ^1^.

	Diets ^2^			
	Experiment 1		Experiment 2	
Item	Prepartum	Postpartum	Prepartum	Postpartum
Dry matter, %	43.2 ± 4.0	50.8 ± 6.0	51.1 ± 1.8	52.6 ± 2.5
	dry matter basis			
Net energy for lactation, ^3^ Mcal/kg	1.56	1.66	1.57	1.67
Crude protein, %	15.5 ± 0.8	17.0 ± 0.4	15.1 ± 0.4	18.1 ± 0.3
Rumen-degradable protein, ^3^ %	9.8	9.2	9.6	9.8
Metabolizable protein, ^3^ %	9.3	11.6	10.4	12.0
Neutral detergent fiber, %	40.2 ± 2.8	34.6 ± 1.5	42.4 ± 4.6	31.0 ± 0.6
Acid detergent fiber, %	34.2 ± 4.6	24.5 ± 1.0	28.3 ± 1.7	22.2 ± 2.4
Lignin, %	5.4 ± 1.4	3.9 ± 0.1	4.9 ± 0.5	3.4 ± 0.2
Nonfibrous carbohydrates, ^4^ %	31.0	33.6	30.7	37.0
Fat, %	5.0 ± 0.2	5.8 ± 0.6	3.9 ± 0.1	7.0 ± 0.9
Ash, %	8.4 ± 0.8	9.0 ± 0.4	7.9 ± 0.6	6.9 ± 0.1
Ca, %	1.11 ± 0.09	0.93 ± 0.06	0.72 ± 0.04	0.67 ± 0.04
P, %	0.31 ± 0.01	0.46 ± 0.02	0.43 ± 0.01	0.47 ± 0.02
K, %	1.60 ± 0.12	1.73 ± 0.11	1.35 ± 0.01	1.70 ± 0.04
Mg, %	0.38 ± 0.01	0.40 ± 0.04	0.35 ± 0.01	0.45 ± 0.01
S, %	0.30 ± 0.05	0.21 ± 0.02	0.21 ± 0.01	0.33 ± 0.01
Na, %	0.25 ± 0.05	0.46 ± 0.01	0.14 ± 0.03	0.37 ± 0.02
Cl, %	0.80 ± 0.04	0.41 ± 0.01	0.31 ± 0.05	0.20 ± 0.01
Zn, mg/kg	54 ± 8	72 ± 20	66 ± 1	60 ± 1
Cu, mg/kg	15 ± 1	14 ± 2	19 ± 1	14 ± 1
Mn, mg/kg	41 ± 5	55 ± 11	58 ± 3	55 ± 5
Dietary cation–anion difference, ^5^ mEq/kg	7.2	216	133	188
Lysine, ^3^ % of metabolizable protein	6.61	6.40	6.86	6.74
Methionine, ^3^ % of metabolizable protein	2.21	2.10	2.23	2.21

^1^ Based on the chemical composition of ingredients analyzed as 3 composite samples per diet. ^2^ Prepartum diets were fed during the last 25 and 22 days of gestation for experiments 1 and 2, respectively; postpartum diets were fed during the first 80 days of lactation. ^3^ Net energy for lactation, rumen-degradable protein, and supply of metabolizable protein, lysine, and methionine were estimated with NRC (2001) based on the nutrient composition of ingredients and the dry matter intake prepartum (12 kg/day in experiment 1; 10.5 kg/day in experiment 2) and postpartum (23 kg/day). ^4^ Nonfibrous carbohydrates = 100 − [crude protein + (neutral detergent fiber − neutral detergent fiber-insoluble crude protein) + fat + ash]. ^5^ Dietary cation–anion difference = (mEq of K + mEq Na) − (mEq S + mEq Cl).

**Table 3:** Nutrient composition of forages (mean ± SD) ^1^.

	Experiment 1 ^2^					Experiment 2 ^2^		
	Corn Silage	A. Silage	Sudan Silage	AHP	AHL	Corn Silage	AHP	AHL
Dry matter %	28.1 ± 1.0	53.0 ± 0.5	27.8 ± 0.7	91.1 ± 0.3	86.5 ± 2.1	33.2 ± 2.4	90.1 ± 0.4	89.7 ± 0.5
	% (dry matter basis)							
CP	9.3 ± 0.6	22.2 ± 0.4	12.1 ± 1.6	18.6 ± 0.2	24.2 ± 1.1	8.4 ± 0.4	19.8 ± 0.2	20.6 ± 2.0
NDF	49.4 ± 0.2	38.2 ± 0.4	56.7 ± 3.4	39.8 ± 0.2	36.6 ± 3.6	45.2 ± 1.6	40.1 ± 0.9	37.8 ± 0.9
ADF	32.4 ± 0.3	29.1 ± 0.5	39.2 ± 2.9	30.3 ± 0.1	29.3 ± 0.9	29.0 ± 1.1	31.4 ± 0.2	29.1 ± 0.8
Lignin	4.3 ± 0.5	6.8 ± 0.8	4.0 ± 1.4	7.9 ± 0.3	7.0 ± 0.7	4.3 ± 0.2	6.9 ± 0.4	6.9 ± 0.1
NDICP ^3^	1.5 ± 0.1	3.6 ± 0.1	1.9 ± 0.3	2.9 ± 0.3	3.9 ± 0.2	1.4 ± 0.1	3.3 ± 0.7	3.3 ± 0.3
Fat	3.9 ± 0.1	3.5 ± 0.1	3.4 ± 0.3	2.7 ± 0.1	2.7 ± 0.1	3.9 ± 0.2	2.9 ± 0.1	2.7 ± 0.2
Ash	5.3 ± 0.5	14.4 ± 0.3	16.0 ± 5.0	8.1 ± 0.4	10.4 ± 0.3	5.1 ± 0.9	8.2 ± 0.7	10.1 ± 0.9
Ca	0.26 ± 0.12	1.38 ± 0.1	0.57 ± 0.11	1.60 ± 0.20	1.78 ± 0.1	0.28 ± 0.05	1.53 ± 0.30	1.65 ± 0.15
P	0.30 ± 0.04	0.31 ± 0.01	0.33 ± 0.03	0.24 ± 0.01	0.36 ± 0.4	0.23 ± 0.01	0.31 ± 0.02	0.36 ± 0.01
K	1.72 ± 0.15	3.35 ± 0.01	2.94 ± 0.26	1.51 ± 0.25	2.97 ± 0.2	0.96 ± 0.06	2.57 ± 0.30	2.60 ± 0.38
Mg	0.15 ± 0.03	0.31 ± 0.01	0.30 ± 0.02	0.46 ± 0.05	0.24 ± 0.03	0.16 ± 0.03	0.31 ± 0.04	0.35 ± 0.05
S	0.12 ± 0.01	0.37 ± 0.01	0.15 ± 0.01	0.35 ± 0.02	0.42 ± 0.07	0.11 ± 0.01	0.30 ± 0.04	0.31 ± 0.01
Na	0.03 ± 0.01	0.41 ± 0.02	0.14 ± 0.05	0.36 ± 0.10	0.20 ± 0.02	0.04 ± 0.02	0.14 ± 0.03	0.19 ± 0.11
Cl	0.40 ± 0.01	0.76 ± 0.04	1.49 ± 0.55	0.92 ± 0.12	1.10 ± 0.13	0.24 ± 0.03	0.61 ± 0.10	0.62 ± 0.15
	mg/kg (dry matter basis)							
Zn	33 ± 5.0	32 ± 4.2	50 ± 14.1	25 ± 3.9	21 ± 0.7	31 ± 3.8	24 ± 3.0	21 ± 2.1
Cu	11 ± 0.7	15 ± 2.1	24 ± 6.4	12 ± 0.8	11 ± 0.7	10 ± 3.1	11 ± 2.0	13 ± 2.1
Mn	27 ± 5.0	54 ± 9.2	59 ± 11.5	25 ± 4.2	62 ± 9.2	39 ± 5.5	36 ± 5.0	35 ± 4.2

^1^ Means of 3 composite samples per ingredient. ^2^ A. = alfalfa; AHP = alfalfa hay prepartum; AHL = alfalfa hay lactation. ^3^ NDICP = neutral detergent-insoluble crude protein.

**Table 4:** Nutrient composition of citrus pulp and grain mixes (mean ± SD) ^1^.

	Experiment 1			Experiment 2		
	Citrus Pulp	Prepartum Mix	Postpartum Mix	Citrus Pulp	Prepartum Mix	Postpartum Mix
Dry matter, %	28.8 ± 4.0	89.1	89.4	29.2 ± 3.4	89.8	90.4
	% (dry matter basis)					
CP	7.5 ± 1.1	25.7	19.9	7.5 ± 1.0	18.7	23.2
NDF	27.7 ± 1.0	22.9	20.1	27.8 ± 0.9	20.9	18.3
ADF	26.7 ± 0.4	9.1	10.7	23.7 ± 3.8	12.6	11.0
Lignin	4.7 ± 2.1	2.2	2.4	4.6 ± 2.0	4.3	3.2
NDICP ^2^	1.1 ± 0.1	7.8	4.6	1.1 ± 0.04	4.8	4.0
Fat	1.7 ± 0.1	10.1	8.8	1.6 ± 0.1	4.9	6.8
Ash	7.7 ± 0.3	16.0	7.1	7.6 ± 0.1	6.2	8.3
Ca	1.92 ± 0.22	1.74	0.59	1.91 ± 0.23	0.31	0.52
P	0.20 ± 0.01	0.87	0.60	0.19 ± 0.02	0.61	0.57
K	1.74 ± 0.13	1.16	0.68	1.70 ± 0.07	1.21	1.12
Mg	0.11 ± 0.01	1.15	0.53	0.11 ± 0.01	0.48	0.55
S	0.09 ± 0.01	1.24	0.27	0.09 ± 0.01	0.37	0.43
Na	0.06 ± 0.01	0.18	0.64	0.06 ± 0.01	0.08	0.57
Cl	0.05 ± 0.01	3.1	0.10	0.06 ± 0.01	0.09	0.08
	mg/kg (dry matter basis)					
Zn	11 ± 3.5	210	82	10 ± 2.8	122	81
Cu	5 ± 0.1	61	16	5 ± 0.7	41	18
Mn	7 ± 1.4	107	50	7.5 ± 2.1	65	31

^1^ Means of 3 composite samples per ingredient. ^2^ NDICP = neutral detergent-insoluble crude protein.

**Table 5:** Effect of feeding rumen-protected choline (RPC) on lactation performance of dairy cows (least squares means and standard error of means, SEM)—experiment 1.

	Treatment ^1^			
Item	Control	RPC	SEM	*p*-Value ^2^
Dry matter intake, kg/day				
Prepartum	11.9	12.5	0.2	0.10
Postpartum	22.2	23.2	0.3	0.06
Milk, kg/day	41.8	43.2	0.6	0.10
3.5% fat-corrected milk, kg/day	42.8	44.8	0.5	0.05
Energy-corrected milk, kg/day	38.5	40.3	0.5	0.04
Milk fat				
%	3.70	3.80	0.04	0.15
kg/day	1.521	1.613	0.025	0.04
Milk true protein				
%	2.81	2.84	0.02	0.43
kg/day	1.166	1.210	0.015	0.08
Milk net energy				
Mcal/kg	0.694	0.705	0.005	0.14
Mcal/day	28.7	30.1	0.4	0.05
Somatic cell score ^2^	1.61	1.66	0.11	0.78
Body condition, scale from 1 to 5				
Change prepartum	−0.17	−0.14	0.03	0.52
Change postpartum	−0.60	−0.52	0.04	0.15
Mean postpartum	3.15	3.24	0.03	0.02

^1^ Cows were fed diets not supplemented (control) or supplemented with 12.9 g/cow/day of choline ion in a rumen-protected form (RPC) from 25 days before to 80 days after calving. ^2^ Log_10_ (somatic cells/12,500)/Log_10_ (2).

## Data Availability

Data are contained within the article. Raw data available upon request to the corresponding author (jepsantos@ufl.edu).

## References

[1] Drackley J.K. (1999). ADSA Foundation Scholar Award. Biology of dairy cows during the transition period: The final frontier?. J. Dairy Sci..

[2] Komaragiri M.V., Erdman R.A. (1997). Factors affecting body tissue mobilization in early lactation dairy cows. 1. Effect of dietary protein on mobilization of body fat and protein. J. Dairy Sci..

[3] Grummer R.R. (1995). Impact of changes in organic nutrient metabolism on feeding the transition dairy cow. J. Anim. Sci..

[4] Arshad U., Zenobi M.G., Staples C.R., Santos J.E.P. (2020). Meta-analysis of the effects of supplemental rumen-protected choline during the transition period on performance and health of parous dairy cows. J. Dairy Sci..

[5] Vance D.E., Vance J.E. (2009). Physiological consequences of disruption of mammalian phospholipid biosynthetic genes. J. Lipid Res..

[6] Imhasly S., Bieli C., Naegeli H., Nystrom L., Ruetten M., Gerspach C. (2015). Blood plasma lipidome profile of dairy cows during the transition period. BMC Vet. Res..

[7] Humer E., Bruggeman G., Zebeli Q. (2019). A meta-analysis on the impact of the supplementation of rumen-protected choline on the metabolic health and performance of dairy cattle. Animals.

[8] Gaal T., Roberts C.J., Reid I.M., Dew A.M., Copp C.M. (1983). Blood composition and liver fat in post parturient dairy cows. Vet. Rec..

[9] Zenobi M.G., Scheffler T.L., Zuniga J.E., Poindexter M.B., Campagna S.R., Castro Gonzalez H.F., Farmer A.T., Barton B.A., Santos J.E.P., Staples C.R. (2018). Feeding increasing amounts of ruminally protected choline decreased fatty liver in nonlactating, pregnant Holstein cows in negative energy status. J. Dairy Sci..

[10] Arshad U., Husnain A., Poindexter M.B., Zimpel R., Nelson C.D., Santos J.E.P. (2023). Rumen-protected choline reduces hepatic lipidosis by increasing hepatic triacylglycerol-rich lipoprotein secretion in dairy cows. J. Dairy Sci..

[11] Arshad U., Santos J.E.P. (2024). Exploring choline’s important roles as a nutrient for transition dairy cows. J. Dairy Sci..

[12] Zom R.L., van Baal J., Goselink R.M., Bakker J.A., de Veth M.J., van Vuuren A.M. (2011). Effect of rumen-protected choline on performance, blood metabolites, and hepatic triacylglycerols of periparturient dairy cattle. J. Dairy Sci..

[13] Elek P., Gaal T., Husveth F. (2013). Influence of rumen-protected choline on liver composition and blood variables indicating energy balance in periparturient dairy cows. Acta Vet. Hung..

[14] Elek P., Newbold J.R., Gaal T., Wagner L., Husveth F. (2008). Effects of rumen-protected choline supplementation on milk production and choline supply of periparturient dairy cows. Animal.

[15] Piepenbrink M.S., Overton T.R. (2003). Liver metabolism and production of cows fed increasing amounts of rumen-protected choline during the periparturient period. J. Dairy Sci..

[16] Zahra L.C., Duffield T.F., Leslie K.E., Overton T.R., Putnam D., LeBlanc S.J. (2006). Effects of rumen-protected choline and monensin on milk production and metabolism of periparturient dairy cows. J. Dairy Sci..

[17] National Research Council (2001). Nutrient Requirement of Dairy Cattle.

[18] Association of Officiating Analytical Chemists (2005). Official Method of Analysis.

[19] Van Soest P.J. (1994). Nutritional Ecology of the Ruminant.

[20] Ferguson J.D., Galligan D.T., Thomsen N. (1994). Principal descriptors of body condition score in Holstein cows. J. Dairy Sci..

[21] Johnson M.M., Peters J.P. (1993). Technical note: An improved method to quantify nonesterified fatty acids in bovine plasma. J. Anim. Sci..

[22] Holm P.I., Ueland P.M., Kvalheim G., Lien E.A. (2003). Determination of choline, betaine, and dimethylglycine in plasma by a high-throughput method based on normal-phase chromatography-tandem mass spectrometry. Clin. Chem..

[23] Jørgensen E., Pedersen A.R. (1998). How to Obtain Those Nasty Standard Errors from Transformed Data—And Why They Should Not be Used.

[24] Davidson S., Hopkins B.A., Odle J., Brownie C., Fellner V., Whitlow L.W. (2008). Supplementing limited methionine diets with rumen-protected methionine, betaine, and choline in early lactation Holstein cows. J. Dairy Sci..

[25] Erdman R.A., Sharma B.K. (1991). Effect of dietary rumen-protected choline in lactating dairy cows. J. Dairy Sci..

[26] Hartwell J.R., Cecava M.J., Donkin S.S. (2000). Impact of dietary rumen undegradable protein and rumen-protected choline on intake, peripartum liver triacylglyceride, plasma metabolites and milk production in transition dairy cows. J. Dairy Sci..

[27] Chung Y.H., Brown N.E., Martinez C.M., Cassidy T.W., Varga G.A. (2009). Effects of rumen-protected choline and dry propylene glycol on feed intake and blood parameters for Holstein dairy cows in early lactation. J. Dairy Sci..

[28] Baldi A., Pinotti L. (2006). Choline metabolism in high-producing dairy cows: Metabolic and nutritional basis. Can. J. Anim. Sci..

[29] Guretzky N.A., Carlson D.B., Garrett J.E., Drackley J.K. (2006). Lipid metabolite profiles and milk production for Holstein and Jersey cows fed rumen-protected choline during the periparturient period. J. Dairy Sci..

[30] Palmquist D.L., Beaulieu A.D., Barbano D.M. (1993). Feed and animal factors influencing milk fat composition. J. Dairy Sci..

[31] Sharma B.K., Erdman R.A. (1988). Abomasal infusion of choline and methionine with or without 2-amino-2-methyl-1-propanol for lactating dairy cows. J. Dairy Sci..

[32] Moore S.J., VandeHaar M.J., Sharma B.K., Pilbeam T.E., Beede D.K., Bucholtz H.F., Liesman J.S., Horst R.L., Goff J.P. (2000). Effects of altering dietary cation-anion difference on calcium and energy metabolism in peripartum cows. J. Dairy Sci..

[33] de Veth M.J., Artegoitia V.M., Campagna S.R., Lapierre H., Harte F., Girard C.L. (2016). Choline absorption and evaluation of bioavailability markers when supplementing choline to lactating dairy cows. J. Dairy Sci..

[34] Chandler T.L., Pralle R.S., Dorea J.R.R., Poock S.E., Oetzel G.R., Fourdraine R.H., White H.M. (2018). Predicting hyperketonemia by logistic and linear regression using test-day milk and performance variables in early-lactation Holstein and Jersey cows. J. Dairy Sci..

